# Noninvasive pressure-strain loop quantitative assessment of left ventricular function in anemic preterm infants with different modes of respiratory support

**DOI:** 10.1007/s10554-024-03138-3

**Published:** 2024-06-04

**Authors:** Ruijie Wang, Hui Yang, Jingbo Jiang, Zhou Lin, Qiuying Zheng, Wei Yu, Shumin Fan, Lei Liu

**Affiliations:** 1 Department of Ultrasound, Shenzhen Children’s Hospital, Shenzhen, China; 2 Department of Neonatal Intensive Care Unit, Shenzhen Children’s Hospital, Shenzhen, China

**Keywords:** Preterm infants, Anemia, Pressure-strain loop, Myocardial work, Echocardiography

## Abstract

To investigate noninvasive pressure-strain loop (PSL) combined with two-dimensional speck tracking imaging and left ventricular pressure measurement in the evaluation of cardiac function changes in anemia of prematurity (AOP) with different modes of respiratory support, and to explore its value in detecting subclinical myocardial injury in preterm infants. This retrospective study included 79 preterm infants with anemia, according to different modes of respiratory support, who were divided into invasive respiratory support group (39 cases) and noninvasive respiratory support group (40 cases). A control group of 40 nonanemic preterm infants with matched age, sex, and gestational age were also included. Complete echocardiography was performed for each included infant. There are PSL parameters that used to evaluate cardiac function, including global longitudinal strain (GLS), global work index (GWI), global constructive work (GCW), global wasted work (GWW), and global work efficiency (GWE) among the three groups were compared. Compared with the control group, the value of GWI, GCW, and GWE were significantly lower and GWW was higher in the AOP groups (*P* < 0.05), and GWI, GCW and GWE were much significantly lower in the invasive respiratory support group than in the noninvasive respiratory support group (*P* < 0.05). There was no significant difference in GLS among the three groups (*P* > 0.05). Noninvasive PSL analysis can quantitatively assess myocardial work in AOP with different respiratory support, which is more sensitive than other conventional echocardiographic indices. This technique may provide a new method for monitoring subclinical myocardial injury with AOP.

## Introduction

Chronic anemia of prematurity (AOP) can cause hypoxia in the heart, brain, and other vital organs, affecting the mode of respiratory support in preterm infants. Early diagnosis of anemia and early detection of changes in cardiac function with timely treatment effectively may improve the development of infants and the quality of long-term survival [1]. The mode of respiratory support is critical in causing pulmonary immaturity and respiratory failure in AOP, which in turn affects systemic oxygen saturation and allows changes in cardiac function [2]. Providing appropriate respiratory support to AOP can help reduce the incidence of respiratory distress syndrome and systemic hypoxemia [3, 4].

The progression of lung disease, as well as the type of respiratory support used contribute to guide decisions about cardiac function interventions [5]. In preterm infants less than 28 weeks gestational age, the need for respiratory support is a pillar of infant survival [6, 7]. Previous studies have shown [8] that preterm infants with older gestational age (28–31 weeks) favored noninvasive respiratory support as well as in-box oxygenation mode. In addition, the mean cardiac troponin T concentration in healthy neonates is 0.087 g/L. Elevated cardiac troponin T may be related to the birth process, with a shift in the respiratory pattern after birth causing functional hypoxemia that may lead to transient myocardial injury [9]. Preterm infants are less tolerant to cardiopulmonary function impairment than term infants, while the compensatory response of cardiac function plays a key role in systemic oxygenation [10]. The time course of postnatal oxygen dependence and mechanical ventilation at different birth ages [8], and parameters of cardiac function in preterm infants at different gestational ages have been previously reported to define bronchopulmonary dysplasia or to determine cardiac function in preterm infants. However, no studies have investigated whether respiratory support in preterm infants differs according to cardiac function.

The pressure-strain loop (PSL) analysis is a new technique for noninvasive assessment of cardiac function. Based on two-dimensional speckle tracking imaging (2D-STI), which depicts the displacement motion of myocardial tissue [11], PSL is constructed by applying strain curves combined with left ventricular pressure to quantify myocardial work, which simultaneously considers both myocardial deformation and cardiac load, overcoming the load dependence of STI and echocardiographic volume parameters for assessing cardiac function.

The PSL analysis was initially used to obtain left ventricular pressures by an invasive method, and Russell et al. [12] first proposed to use brachial artery pressure measured by a cuffed sphygmomanometer at rest instead of left ventricular pressures obtained by an invasive method, according to their results, significant correlation was confirmed by clinical studies (*r* = 0.99). In recent years, several studies have shown [13, 14] that quantitative calculation of myocardial work by noninvasive PSL analysis more accurately reflected myocardial motion and can detect small changes in left ventricular function at an early stage. At present, there are few reports on the application of this technique in newborns [15]. The purpose of this study was to apply the PSL analysis to quantitatively calculate myocardial work to assess changes in left ventricular function in AOP with different respiratory support modalities, and to investigate the value of the PSL analysis in the clinical setting to detect subclinical myocardial injury in preterm infants.

## Methods

### Study population

This was a retrospective study. The study population consisted of 106 AOP hospitalized in the Neonatal Intensive Care Unit of Shenzhen Children’s Hospital between October 2021 and October 2022, and 79 cases had complete records of respiratory support parameters. Among them, 39 AOP patients received mechanical ventilation support through trachea (AOP Invasive group), 40 AOP patients inhaled oxygen through face mask, head mask or low flow nasal intubation, (b) nasal continuous positive airway pressure ventilation through nasal continuous positive airway pressure, high flow nasal intubation, and (c) inhaled oxygen autonomously through box oxygen inhalation (AOP Noninvasive group). 40 nonanemic preterm infants matched for age, sex, and gestational age were selected as the control group.

Inclusion criteria for the AOP and control groups were as follows: gestational age < 37 weeks; adherence to the British Committee for Standards in Haematology anemia index for preterm infants; complete clinical records and echocardiographic data. The inclusion criteria for the control group were as follows: gestational age < 37 weeks; not meeting the criteria for anemia; complete clinical records and echocardiographic data. Exclusion criteria for the AOP and control groups were as follows: congenital heart disease [except patent foramen ovale (PFO)]; hemodynamically significant patent ductus arteriosus (hsPDA); receipt of cardiac medications or diuretics; severe pneumonia; severe infection; hypoglycemia; hyperbilirubinemia; ABO hemolytic anemia; history of red blood cell transfusion; neonatal hemorrhagic disorders (e.g., pulmonary hemorrhage, gastrointestinal bleeding, etc.); and prenatal and delivery hemorrhagic disorders.

Clinical baseline data were collected for all participants, including sex, gestational age, corrected gestational age, age, body length, body weight, blood pressure, respiratory support parameters, hemoglobin (HB), and hematocrit (HCT). Written informed consent was obtained from all participants, and the study was approved by the ethics committee of our hospital [Shenzhen Children’s Hospital Medical Ethics Review (Scientific Research) No. 2,022,120].

### Echocardiography

A complete transthoracic echocardiogram was performed under resting or sleeping conditions using Vivid E95 ultrasound system (General Electric Vingmed Ultrasound, Milwaukee, WI, USA) 6 S-D probe to obtain the best image quality of 70–80 frames per second. All parameters were averaged over three consecutive cardiac measurement cycles. Image acquisition was performed by ultrasound doctors unaware of the study content and clinical data, all in strict accordance with the echocardiography operation standard of American Society of Echocardiography (ASE). Left ventricular end-diastolic diameter (LVEDD), left ventricular end-systolic diameter (LVESD), interventricular septum thickness in end-diastole (IVSD), and posterior wall thickness (PWT) were measured by M-mode echocardiography; left ventricular end-diastolic volume (LVEDV), left ventricular end-systolic volume (LVESV), stroke volume (SV), cardiac output (CO), and heart rate (HR) were obtained by apical 4-chamber view three dimensional echocardiography; early diastolic peak velocity (E) of mitral valve was measured by pulsed wave Doppler imaging; peak early diastolic velocity of mitral annulus (e’) was measured by tissue Doppler imaging.

Brachial blood pressure of the right upper arm was measured using the noninvasive blood pressure cuff mode of the bedside ECG monitor when the heart rate was stable for 20 min and without limb movement for 10 min before echocardiography, which obtained systolic blood pressure (SBP) and diastolic blood pressure (DBP). The respiratory support parameters such as fractional concentration of inspired oxygen and mean airway pressure were recorded, and the mean values of three measurements were taken for statistical analysis.

### Analysis of strain and myocardial work

**Fig. 1:**
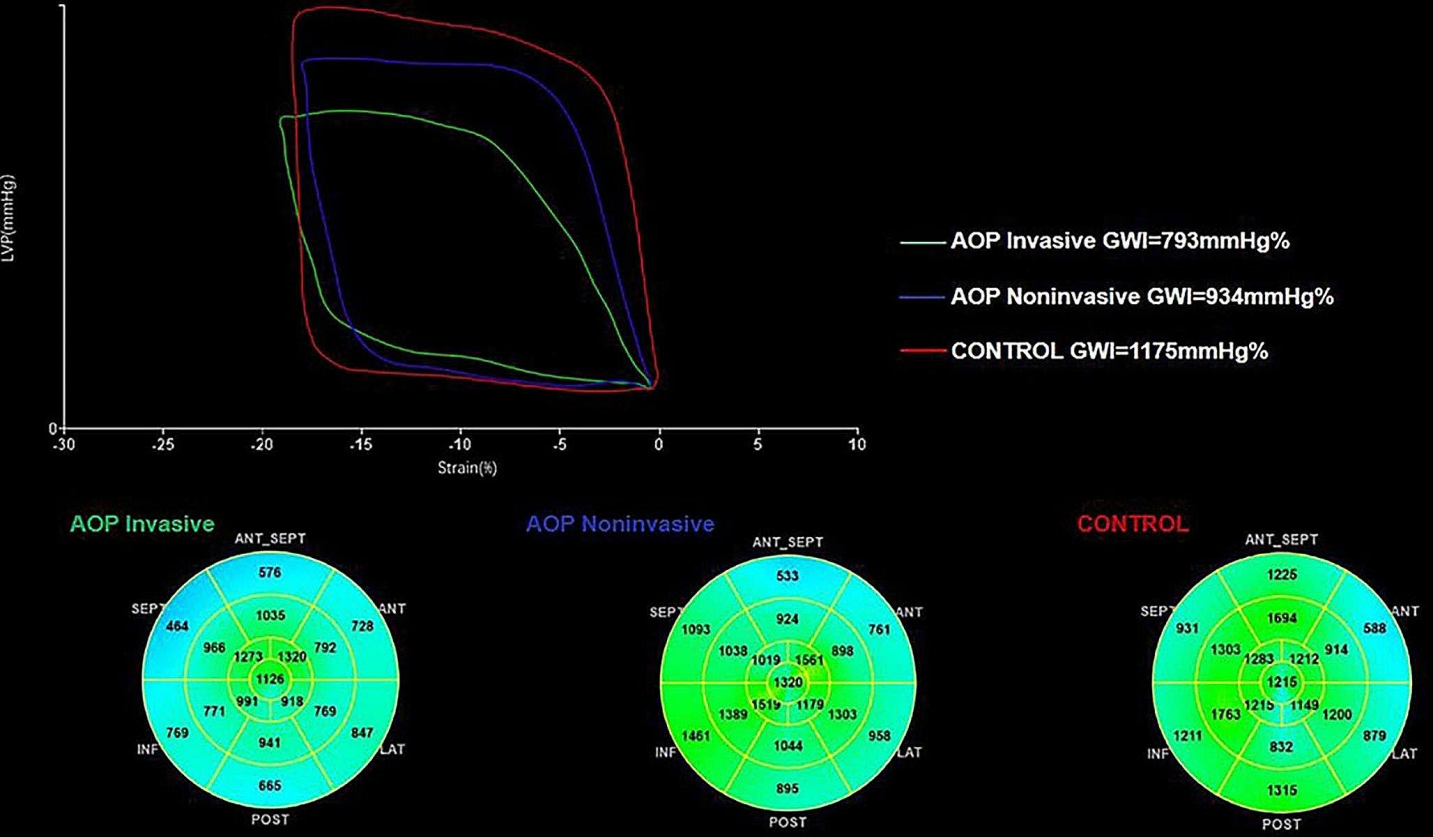
Pressure-strain loop diagram

Dynamic images of three consecutive cardiac cycles from apical three-chamber, apical four-chamber, and apical two-chamber images were applied, and the stored raw data were imported into offline software (EchoPAC PC version 203; GE Healthcare, Horten, Norway) to measure the data. The endocardial and epicardial borders were tracked in the dynamic images using the automatic imaging function, and the area of interest was adjusted by manually correcting the endocardial border or width if necessary, and the global longitudinal strain (GLS) was obtained for 17 segments of the left ventricular myocardium. Input brachial artery blood pressure, and choose mitral and aortic valve opening and closing time points on apical three-chamber dynamic images. The software obtains noninvasive PSL by integrating GLS, blood pressure, and valve opening and closing times. The annular area surrounded by PSL, the product of two-dimensional strain and ventricular pressure, represents the total work done from mitral valve closure to mitral valve opening, represented by global work index (GWI) (Fig. 1). Meanwhile, other parameters of myocardial work were calculated as follows: global constructive work (GCW, mmHg%), which includes the sum of the work done by systolic myocardial shortening and isovolumic diastolic myocardial stretching, that is, the work done by the myocardium contributing to ventricular systolic ejection; global wasted work (GWW, mmHg%), which includes the sum of work done by systolic myocardial stretch and isovolumic diastolic myocardial shortening, work that does not contribute to ventricular systolic ejection; global work efficiency (GWE, %), calculated as GWE=GCW/(GCW + GWW)

The figure shows three example of the pressure strain loops and the 17 segments of myocardial work, including invasive and noninvasive respiratory support group and control group. On the top left, the area under the loop represents GWI, and the lower shows the 17-segment bull’s-eye view of the GWI; the higher the GWI, the closer the color is to green, and vice versa, the closer it is to blue. *AOP Invasive* invasive respiratory support group, *AOP Noninvasive* noninvasive respiratory support group, *GWI* global work index.

### Statistical analysis

Statistical analysis was performed using SPSS 28.0 (SPSS version 28.0; IBM Corp., Armonk, NY, USA), and graphs were presented using GraphPad Prism (version 7.0.5). Means ± standard deviations (x ± s) and 95% confidence intervals (CI) were used to represent measures that conformed to a normal distribution with equal variance. Medians (upper and lower quartiles) were used to represent measures that did not conform to a normal distribution. The number of cases (proportions) [n (%)] was used to represent count data. Continuous variables were analyzed by T test or Mann-Whitney U test. 15 patients in the AOP group and 15 in the control group were randomly selected for repeated testing. Bland-Altman analysis was used to test the interobserver and intraobserver consistency of myocardial work parameters. Interobserver consistency was assessed by two experienced ultrasound physicians after independent measurements, and intraobserver consistency was assessed by one ultrasound physician after two measurements. Two-sided *P* < 0.05 was considered statistically significant.

## Results

### Study subjects

There were no significant differences between the AOP group and the control group in terms of sex, gestational age, corrected gestational age, age, length, weight, number of PFO cases, and number of PDA cases (*P* > 0.05; Table 1). HB, HCT, and oxygenation index (OI) were lower, while heart rate and fractional concentration of inspired oxygen (FiO_2_) was higher, which was statistically significant (*P* < 0.05).

Compared to the noninvasive respiratory support group, HB, HCT, SBP, DBP, and OI of the invasive respiratory support group were lower, while FiO_2_ and mean airway pressure (PAW) were higher, and the differences were statistically significant (*P* < 0.05; Table 2).

**Table 1:** Baseline characteristics of AOP and control group

	AOP (*n* = 79)	Control (*n* = 40)	*P* value
Males, n(%)	51 (65%)	28 (70%)	0.553
GA(w)	27.976 ± 2.130	28.657 ± 1.885	0.090
CGA(w)	30.872 ± 2.667	31.568 ± 1.990	0.148
Day(d)	17.0 (7.0, 30.5)	17.0 (14.0, 27.0)	0.363
Current length(cm)	39.506 ± 2.813	40.700 ± 4.468	0.179
Current weight(g)	1.471 ± 0.523	1.590 ± 0.413	0.215
PFO: n(%)	64 (81%)	29 (73%)	0.288
PDA: n(%)	31 (39%)	15 (38%)	0.854
HB(g/l)	93 (84, 104)	123 (109, 150)	< 0.001*
HCT(%)	27.9 (24.7, 31.0)	39.8 (35.1, 47.0)	< 0.001*
SBP(mmHg)	69.0 (54.5, 75.0)	70.5 (65.8, 72.0)	0.300
DBP(mmHg)	38.0 (30.0, 42.0)	40 (35.0, 46.0)	0.084
HR(bpm)	160.716 ± 15.208	143.875 ± 13.074	< 0.001*
PaO2(mmHg)	93.538 ± 3.950	94.288 ± 2.709	0.283
FiO_2_(%)	25.861 ± 4.629	23.925 ± 2.495	< 0.001*
PAW(cmH_2_O)	7.383 ± 1.464	7.115 ± 0.842	0.008
OI(mmHg)	344.6 (320.8, 438.1)	451.7 (438.3, 457.1)	< 0.001*

*P < 0.05, significantly different compared with Control

*GA* gestational age, *CGA* corrected gestational age, *PFO* patent foramen ovale, *PDA* patent ductus arteriosus, *HB* hemoglobin, *HCT* hematocrit, *SAP* systolic blood pressure, *DAP* diastolic blood pressure, *HR* heart rate, *PaO2* arterial partial pressure of oxygen, *FiO2* fractional concentration of inspired oxygen, *PAW* mean airway pressure, *OI* oxygenation index

**Table 2:** Baseline characteristics and echocardiographic parameters of AOP with invasive and noninvasive respiratory support

	Invasive (*n* = 39)	Noninvasive (*n* = 40)	*P* value
Males, n(%)	26 (67%)	25 (63%)	0.699
GA(w)	27.681 ± 1.893	28.253 ± 2.325	0.226
CGA(w)	30.648 ± 2.512	31.089 ± 2.824	0.466
Day(d)	16.0 (8.0, 30.0)	18.0 (7.0, 31.3)	0.953
Current length(cm)	39.000 ± 3.000	40.000 ± 2.560	0.075
Current weight(g)	1.436 ± 0.495	1.505 ± 0.554	0.559
PFO: n(%)	31 (79%)	33 (83%)	0.733
PDA: n(%)	18 (46%)	13 (33%)	0.214
HB(g/l)	85.0 (78.5, 94.0)	99.5 (92.0, 107.5)	< 0.001*
HCT(%)	25.4 (22.9, 28.6)	30.0 (27.6, 32.4)	< 0.001*
SBP(mmHg)	59.0 (48.0, 68.5)	72.5 (69.0, 76.0)	< 0.001*
DBP(mmHg)	32.0 (25.5, 38.0)	41.0 (38.0, 43.3)	< 0.001*
HR(bpm)	165.538 ± 13.928	156.015 ± 15.087	0.005*
PaO2(mmHg)	93.279 ± 4.292	93.790 ± 3.623	0.569
FiO_2_(%)	27.308 ± 4.900	24.450 ± 3.915	0.005*
PAW(cmH_2_O)	8.474 ± 0.716	6.320 ± 1.199	< 0.001*
OI(mmHg)	343.2 (313.0, 388.0)	420.2 (326.3, 457.3)	0.006*

*P < 0.05, significantly different compared with Noninvasive group

### Standard echocardiographic parameters and myocardial work analysis

Compared to the control group, CO were higher in the AOP group, and the differences were statistically significant (*P* < 0.05; Table 3). There was no significant difference in standard echocardiographic parameters between the noninvasive and invasive respiratory support groups (*P* > 0.05; Table 4).

Compared to the control group, GWI, GCW, and GWE were significantly lower and GWW was significantly higher in the AOP group, and the differences were statistically significant (*P* < 0.05; Table 3). Compared to the noninvasive respiratory support group, GWI, GCW, and GWE were significantly lower in the invasive respiratory support group, and the differences were statistically significant (*P* < 0.05; Table 4); GWW had a tendency to increase, but the differences were not statistically significant (*P* > 0.05). The difference of GLS among groups was not statistically significant (*P* > 0.05).

**Table 3:** Echocardiographic parameters and myocardial work of AOP and control group

	AOP (*n* = 79)	Control (*n* = 40)	*P* value
*Standard Ultrasound*			
LVEDD(mm)	16.667 ± 2.671	14.825 ± 2.194	0.519
LVESD(mm)	10.140 ± 1.821	9.375 ± 1.690	0.695
IVSD(mm)	3.086 ± 0.573	3.143 ± 0.422	0.544
PWD(mm)	2.828 ± 0.549	2.878 ± 0.340	0.901
LVEDV(ml)	5.190 ± 0.948	4.538 ± 1.067	0.669
LVESV(ml)	1.736 ± 0.411	1.575 ± 0.385	0.848
SV(ml)	3.453 ± 0.625	2.963 ± 0.816	0.607
CO(L/min)	0.556 ± 0.118	0.422 ± 0.109	< 0.001*
LVEF(%)	66.665 ± 3.650	65.989 ± 5.599	0.584
FS(%)	0.392 ± 0.057	0.367 ± 0.076	0.796
Tei index	0.471 ± 0.064	0.414 ± 0.115	0.492
E/e’ ratio	11.3 (8.5, 13.8)	11.0 (9.0, 14.0)	0.868
GLS	-18.722 ± 1.987	-18.725 ± 1.467	0.992
*Myocardial Work*			
GWI	754.0 (649.0, 910.0)	995.5 (847.3, 1128.5)	< 0.001*
GCW	940.0 (801.0, 1065.0)	1134.5 (976, 1247.8)	< 0.001*
GWW	76.0 (55.5, 97.0)	55.5 (44.8, 77.3)	0.002*
GWE	92.063 ± 2.657	94.650 ± 2.020	0.002*

*P < 0.05, significantly different compared with Control group *LVEDD* left ventricular end-diastolic internal diameter, *LVESD* left ventricular end-systolic internal diameter, *IVSD* end-diastolic internal septal thickness, *PWT* left ventricular posterior wall thickness, *LVEDV* left ventricular end-diastolic volume, *LVESV* left ventricular end-systolic volume, *SV* cardiac output per beat, *CO* cardiac output, *E/e’* ratio of early diastolic peak velocity of mitral valve to early peak diastolic velocity of mitral annulus, *GLS* global longitudinal strain, *GWI* global work index, *GCW* global constructive work, *GWW* global wasted work, *GWE* global work efficiency

**Table 4:** Echocardiographic parameters and myocardial work of AOP with invasive and noninvasive respiratory support

	Invasive (*n* = 39)	Noninvasive (*n* = 40)	*P* value
*Standard Ultrasound*			
LVEDD(mm)	16.331 ± 2.608	17.194 ± 2.724	0.273
LVESD(mm)	9.997 ± 1.934	10.280 ± 1.718	0.494
IVSD(mm)	3.018 ± 0.570	3.153 ± 0.575	0.300
PWD(mm)	2.815 ± 0.514	2.841 ± 0.587	0.836
LVEDV(ml)	5.114 ± 0.809	5.264 ± 1.071	0.485
LVESV(ml)	1.722 ± 0.311	1.750 ± 0.492	0.761
SV(ml)	3.392 ± 0.577	3.513 ± 0.671	0.391
CO(L/min)	0.563 ± 0.113	0.549 ± 0.123	0.615
LVEF(%)	66.289 ± 3.174	67.032 ± 4.067	0.369
FS(%)	0.388 ± 0.069	0.395 ± 0.042	0.635
Tei index	0.481 ± 0.073	0.463 ± 0.054	0.218
E/e’ ratio	11.0 (8.3, 13.9)	11.7 (9.0, 13.8)	0.776
GLS	-18.974 ± 2.006	-18.475 ± 1.961	0.267
*Myocardial Work*			
GWI	695.0 (616.5, 839.0)	827.0 (714.3, 932.8)	0.008*
GCW	842.0 (749.5, 978.5)	979.5 (859.0, 1072.8)	0.017*
GWW	80.0 (61.0, 97.5)	67.0 (51.3, 92.8)	0.184
GWE	91.462 ± 2.222	92.725 ± 2.873	0.020*

*P < 0.05, significantly different compared with Noninvasive group

### Repeatability of myocardial work

**Fig. 2:**
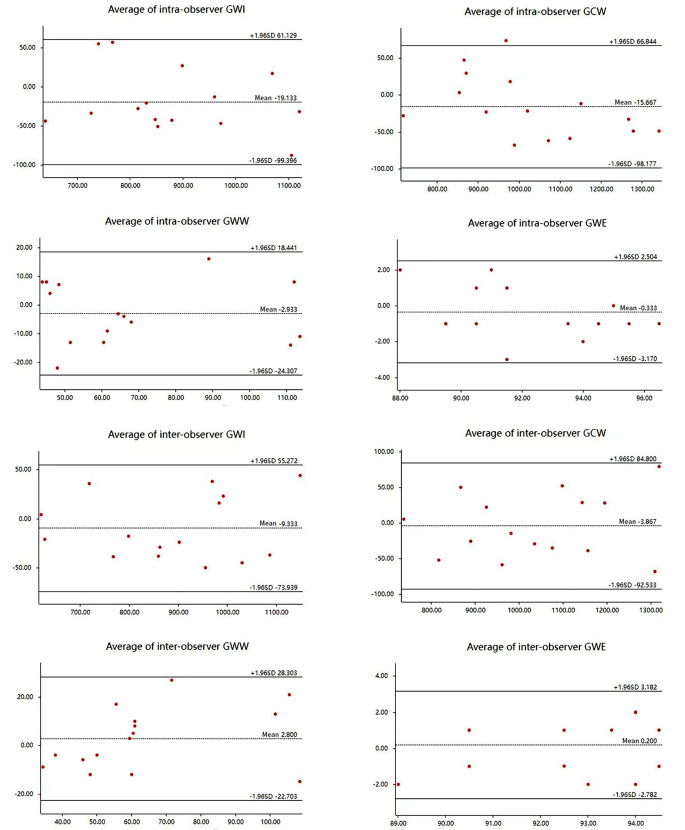
Bland-Altman Analysis of Myocardial Work Parameters. Bland − Altman plots respectively of intra-observer agreement for: GWI, GCW, GWW, and GWE; Bland − Altman plots respectively of inter-observer agreement for: GWI, GCW, GWW, and GWE. *GWI* global work index, *GCW* global constructive work, *GWW* global wasted work, *GWE* global work efficiency

All parameters of myocardial work showed good intra- and interobserver correlations.Bland-Altman plot analysis showed good inter- and intraobserver repeatability and agreement in the analysis of various parameters of myocardial work (Fig. 2).

## Discussion

Anemia in preterm infants is a pathological condition characterized by altered hemodynamics. Anemia leads to tissue hypoxia, which activates compensatory mechanisms that initially compensate for CO by increasing myocardial contraction, resulting in increased SV and heart rate and increased sympathetic activity to maintain adequate oxygen delivery [16]. In anemia, blood viscosity decreases and increased venous return leads to increased cardiac preload and decreased peripheral vascular resistance leads to decreased cardiac afterload [17]. Therefore, in order to balance the benefits and risks of cardiopulmonary function diagnosis and treatment of premature infants, early evaluation of cardiac function changes of AOP with different respiratory support by PSL analysis is of great value in clinical diagnosis and treatment.

AOP with different respiratory support all experienced post-anemic compensatory response, higher LVEDD and cardiac output than the control group, and reduced myocardial work. Cardiac function was more severely impaired in the invasive group, and work efficiency was significantly reduced. Noninvasive respiratory support favored oxygen transport or utilization efficiency. The higher LVEDD and CO of the noninvasive group which indicated that the preterm infants with the noninvasive respiratory support had greater anemic compensatory response than those with the invasive respiratory support. Despite the increase of CO due to the compensatory cardiac response, the systolic function was already damaged to some extent.

In this study, the parameters widely used to evaluate left ventricular systolic function, including LVEF, FS and GLS, were within the normal range and had no significant changes, suggesting that the above indices are not very sensitive for early diagnosis of myocardial injury, which is consistent with the results of previous results of Cui [18] and others. It is considered that the disease may not cause heart failure when the disease is involved in the heart, resulting in a mild decrease in left ventricular systolic function and is not recognized.

Noninvasive myocardial work parameters to assess cardiac function in AOP may provide early and more sensitive detection of subclinical myocardial injury, which is particularly important in AOP because cardiac function may change with the course of anemia, changes in respiratory support, drug use and nursing care. The inclusion of myocardial work parameters in the echocardiographic evaluation of AOP may serve as a tool for clinicians to assess cardiac function, and early detection of altered cardiac function in AOP may guide early treatment of preterm infants with asymptomatic left ventricular systolic dysfunction.

### Limitations

First of all, in this study, the average systolic blood pressure of brachial artery measured by non-invasive cuff measurement rather than invasive measurement was used to evaluate the peak left ventricular pressure. Although this method has shown good correlation with invasive measurements in most previous studies (*r* = 0.99), the results of the two methods are not completely consistent. In addition, the high fluctuation of blood pressure, respiratory support mode and mean airway pressure parameters are highly variable in preterm infants, so parameters should be accurately recorded at steady state. Finally, this study is a single-center, small sample study, and studies with larger sample sizes are needed to be clarified to help neonatologists assess cardiac function of AOP and accurately evaluate the status of cardiopulmonary function in preterm infants.

## Conclusion

The application of noninvasive PSL analysis is sensitive to the early detection of subclinical myocardial injury in AOP and is more sensitive than previous cardiac function indicators in assessing the differences in cardiac function in AOP with different respiratory patterns. This is a valuable technique that can be used as a reference to guide the treatment plan to improve cardiopulmonary function in AOP.

## References

[1] German KR, Juul SE (2023). Neonatal Anemia. Curr Pediatr Reviews.

[2] Glaser K, Wright CJ (2021). Indications for and risks of noninvasive respiratory support. Neonatology.

[3] Welsford M, Nishiyama C, Shortt C, Weiner G, Roehr CC, Isayama T (2019). Initial oxygen use for Preterm Newborn Resuscitation: a systematic review with Meta-analysis. Pediatrics.

[4] Madar J, Roehr CC, Ainsworth S, Ersdal H, Morley C, Rudiger M (2021). European Resuscitation Council guidelines 2021: newborn resuscitation and support of transition of infants at birth. Resuscitation.

[5] Kelly LE, Shah PS, Hakansson S, Kusuda S, Adams M, Lee SK (2017). Perinatal health services organization for preterm births: a multinational comparison. J Perinatol.

[6] Norman M, Jonsson B, Wallström L, Sindelar R (2022). Respiratory support of infants born at 22–24 weeks of gestational age. Semin Fetal Neonatal Med.

[7] Patel RM, Kandefer S, Walsh MC, Bell EF, Carlo WA, Laptook AR (2021). Survival and causes of death in extremely preterm infants in the Netherlands. Archives of disease in childhood. Fetal Neonatal Ed.

[8] Norman M, Jonsson B, Söderling J, Björklund LJ, Håkansson S (2023). Patterns of respiratory support by gestational age in very Preterm infants. Neonatology.

[9] Awada H, Al-Tannir M, Ziade MF, Alameh J, El-Rajab M (2007). Cardiac troponin T: a useful early marker for cardiac and respiratory dysfunction in neonates. Neonatology.

[10] Merve H, Ayhan P, Selcuk G, Mehmet B (2021). How are cardiac functions altered in pediatric patients receiving oral iron supplementation due to anemia?. J Sci Perspect.

[11] Castaldi B, Bordin G, Favero V, Nardo D, Previati F, Salvadori S (2018). Early modifications of cardiac function in preterm neonates using speckle tracking echocardiography. Echocardiography (Mount Kisco N Y).

[12] Russell K, Eriksen M, Aaberge L, Wilhelmsen N, Skulstad H, Remme E (2012). A novel clinical method for quantification of regional left ventricular pressure-strain loop area: a non-invasive index of myocardial work. Eur Heart J.

[13] Ren F, Xue T, Tang G, Zhang M, Zhao J, Chen Y (2022). Assessment of Myocardial Work of the left ventricle before and after PCI in patients with Non-ST-Segment elevation Acute Coronary syndrome by pressure-strain Loop Technology. Comput Math Methods Med.

[14] Jaglan A, Roemer S, Khandheria B (2020). Myocardial work index: it works. European heart journal. Cardiovasc Imaging.

[15] Yanase Y, Iwashima S, Takahashi K (2022). Echocardiographic reference ranges of non-invasive myocardial work indices in newborns. Circulation Rep.

[16] Cibulskis CC, Maheshwari A, Rao R, Mathur AM (2021). Anemia of prematurity: how low is too low?. J Perinatology: Official J Calif Perinat Association.

[17] Saleemi MS, Bruton K, El-Khuffash A, Kirkham C, Franklin O, Corcoran JD (2013). Myocardial assessment using tissue doppler imaging in preterm very low-birth weight infants before and after red blood cell transfusion. J Perinatology: Official J Calif Perinat Association.

[18] Cui C, Liu L, Li Y, Liu Y, Huang D, Hu Y (2020). Left ventricular pressure-strain Loop-based quantitative examination of the Global and Regional Myocardial Work of patients with dilated cardiomyopathy. Ultrasound Med Biol.

